# Relationships between accelerometry and general compensatory movements of the upper limb after stroke

**DOI:** 10.1186/s12984-020-00773-4

**Published:** 2020-10-20

**Authors:** Jessica Barth, Joeseph W. Klaesner, Catherine E. Lang

**Affiliations:** 1 Washington University School of Medicine, Program in Physical Therapy, St. Louis, MO USA; 2 Washington University School of Medicine, Program in Occupational Therapy, St. Louis, MO USA; 3 Department of Neurology, Washington University School of Medicine, St. Louis, MO USA; 4 Department in Biomedical Engineering, Washington University, St. Louis, MO USA

**Keywords:** Accelerometry, Stroke rehabilitation, Upper extremity, Cerebrovascular disease

## Abstract

**Background:**

Standardized assessments are used in rehabilitation clinics after stroke to measure restoration versus compensatory movements of the upper limb. Accelerometry is an emerging tool that can bridge the gap between in- and out-of-clinic assessments of the upper limb, but is limited in that it currently does not capture the quality of a person’s movement, an important concept to assess compensation versus restoration. The purpose of this analysis was to characterize how accelerometer variables may reflect upper limb compensatory movement patterns after stroke.

**Methods:**

This study was a secondary analysis of an existing data set from a Phase II, single-blind, randomized, parallel dose–response trial (NCT0114369). Sources of data utilized were: (1) a compensatory movement score derived from video analysis of the Action Research Arm Test (ARAT), and (2) calculated accelerometer variables quantifying time, magnitude and variability of upper limb movement from the same time point during study participation for both in-clinic and out-of-clinic recording periods.

**Results:**

Participants had chronic upper limb paresis of mild to moderate severity. Compensatory movement scores varied across the sample, with a mean of 73.7 ± 33.6 and range from 11.5 to 188. Moderate correlations were observed between the compensatory movement score and each accelerometer variable. Accelerometer variables measured out-of-clinic had stronger relationships with compensatory movements, compared with accelerometer variables in-clinic. Variables quantifying time, magnitude, and variability of upper limb movement out-of-clinic had relationships to the compensatory movement score.

**Conclusions:**

Accelerometry is a tool that, while measuring movement quantity, can also reflect the use of general compensatory movement patterns of the upper limb in persons with chronic stroke. Individuals who move their limbs more in daily life with respect to time and variability tend to move with less movement compensations and more typical movement patterns. Likewise, individuals who move their paretic limbs less and their non-paretic limb more in daily life tend to move with more movement compensations at all joints in the paretic limb and less typical movement patterns.

## Introduction

As advances in medicine persist, more people are surviving a stroke. Over 80% of those affected will have persistent hemiparesis of their upper limb [1]. These people will be left with chronic disability when trying to complete their activities of daily living (ADL), and an even larger number will not resume their normal daily activities completed prior to stroke [2]. At this time, physical and occupational therapy is the only option available to improve upper limb use after stroke. The ultimate goal of these therapies is to restore the use of the upper limb to the same level it was used before the stroke. Most individuals, however, only partially regain function of their upper limb requiring compensations of the upper limb to complete daily tasks. The differentiation between restoration of upper limb movement and compensation is an area of high interest in stroke rehabilitation [3]. Compensation can occur on multiple levels, such as using an alternative movement pattern, using an alternative tool or support (e.g. built up spoon for self-feeding), and/or using an alternate means to achieve the task (e.g. completion of an activity by a spouse rather than the individual). For the purposes of this paper, compensatory movements will refer to completion of the same movement but with an alternative movement pattern. Specifically, this level of compensatory movements typically describe accessory movements of the head, trunk and upper limb that an individual incorporates in order to accomplish tasks. A simple example is that if an individual lacks shoulder flexion, or the ability to raise their arm in front of them, the individual lifts their arm by raising it more to the side and bending forward with the trunk [4, 5]. Many in the neurorehabilitation field view compensation and restoration as a dichotomy, where individuals will either be classified as using compensatory movement patterns or restored movement patterns. Return of upper limb function may be better conceptualized as a gradient, with individuals having degrees of compensatory movement patterns [6].

Currently, many in-clinic standardized assessments have some aspects that measure use of compensatory movement patterns. For example, the Reaching Performance Scale specifically assesses compensatory movements of the upper limb during reaching in people with hemiparesis [7]. The Wolf Motor Function Test’s Functional Ability Scale reduces scores if movement compensations were observed during item completion [8]. The Fugl-Meyer arm motor scale, an impairment scale, focused on movement patterns, takes points off where specific compensatory movements are observed on each item [9]. Additionally the Action Research Arm Test (ARAT) scores individuals completing functional reach to grasp tasks with consideration of the quality of the reach and grasp pattern along with the fluidity or precision of the task [10, 11]. Standardized assessments have the ability to measure upper limb functional capacity and compensatory movements of the upper limb after stroke, however these assessments only capture one piece of upper limb recovery after stroke.

The current gold standard in the field to measure quality of movement or compensatory movements is through the use of 3D kinematics [12]. Kinematics provides the most detailed assessment of how an individual moves after stroke. It is not realistic, however, to use kinematics in the clinic for all patients due to cost of equipment, time required to test, and training of personnel. This leaves standardized assessments to be the alternative and most accessible measure of compensatory movement patterns. This gap in measurement has lead our lab to question how we might utilize our existing accelerometry methodology to capture some of these changes in compensatory movement.

In-clinic assessments are limited in that they measure the individual’s ability to use the limb in a standardized, structured setting, leaving the individuals actual activity of the limb during daily life unaccounted for. Over the past 5 years, methodology has been developed to measure upper limb activity in daily life using wearable sensors (accelerometers) [13, 14]. Accelerometry can quantify how much and how often a person uses their affected limb during their daily life, bridging the gap between in and out-of-clinic assessment. Current accelerometer metrics quantify time, magnitude and variability of movement of the upper limb [15–19]. A limitation of current accelerometry methods is that they quantify the amount of movement, but do not capture the quality of a person’s movement, an important concept to assess compensation versus restoration.

The purpose of this secondary analysis was to characterize how accelerometer variables reflect upper limb compensatory movement patterns after stroke. Relationships between compensatory movement patterns and accelerometer variables were calculated for both in-clinic and out-of-clinic time points. Both time points were included as the in-clinic time includes completion of standardized assessments and participation in an intensive upper limb therapy protocol. Due to the nature of the therapy protocol, we anticipated there may be different relationships because during the in-clinic time participants are intentionally training their affected limb. The out-of-clinic recordings captures the individual in their free-living environment, providing a more realistic picture of how the individual uses their upper limb in daily life. It is hypothesized that quantitative metrics from accelerometers both in and out-of-clinic will have moderate associations with compensatory movement patterns of the upper limb.

## Methods

This study was a secondary analysis of an existing data set from a Phase II, single-blind, randomized, parallel dose–response trial (NCT0114369) [20]. Sources of data utilized were: (1) a compensatory movement score derived from video analysis of the Action Research Arm Test (ARAT), and (2) calculated accelerometer variables from the same time point during study participation.

### Participants

Inclusion criteria were (1) ischemic or hemorrhagic stroke as determined by neurologist and consistent with neuroimaging; (2) time since stroke ≥ 6 months; (3) cognitive skills to actively participate, as indicated by scores of 0–1 on items 1b and 1c of the National Institutes of Health Stroke Scale (NIHSS); (4) unilateral upper limb weakness, as indicated by a score of 1–3 on items 5 (arm item) on the NIHSS; and (5) mild-to-moderate functional motor capacity of the paretic upper limb, as indicated by a score of 10–48 on the ARAT [10, 11]. Exclusion criteria were (1) participant unavailable for 2 month follow-up (2) inability to follow-2-step commands; (3) psychiatric diagnoses; (4) current participation in other UL stroke treatments (ex/Botox); (5) other neurological diagnoses; (6) participants living further than 1 h away or were unwilling to travel for assessments and treatment sessions; and (7) pregnancy. The clinical trial was approved by the Washington University Human Research Protection Office and all subjects provided informed consent prior to trial participation.

### Compensatory Movement Score

A compensatory movement score was derived from video recordings of available baseline or subsequent ARATs. We first developed a checklist to quantify the degree of movement compensations of the upper limb. Compensatory movement information was synthesized from nine standardized assessments of the upper limb measuring quality of movement or compensatory movement patterns [7, 8, 10, 21–26]. Descriptions of compensatory movement patterns of the upper limb were extracted from the assessments and organized to generate the list of items on the checklist. The checklist was piloted and refined following feedback from licensed physical and occupational therapists. The Additional file 1: Table provides the final checklist.

Items selected for the checklist were compensatory behaviors specific to each joint. Compensatory behaviors on the checklist [7, 8, 10, 21–26] included movements at the head, trunk, shoulder, elbow, forearm, wrist, fingers, and fluidity/movement precision. The administration of the ARAT adhered to the standardized instructions recommended by Yozbatiran et al. [10]; participants were not provided with instructions regarding how the task should be completed. Compensations were scored as present (+ 1 point) or absent (0 points) from the videotaped completion of the ARAT. For example, potential trunk compensations could be: excessive trunk flexion or excessive trunk side bending/rotation. In addition to compensations at each joint, an item labeled fluidity and precision of moment was added to capture jerky or uncoordinated sub-movements and multiple attempts to complete a task [7, 8, 10, 21–26].

Raters were current physical therapy students and one undergraduate summer intern. Non-licensed individuals were selected to decrease bias. In piloting, we found that licensed therapists tended to rate compensatory movement scores higher due to anticipation of expected movement patterns, whereas students simply rated if a compensatory movement was present or absent. Raters were trained prior to beginning scoring videos for data collection. Raters were provided with a manual that described the movement compensations. Then, raters scored a video side-by-side with a trainer (JB), where they discussed and highlighted each type of movement compensation. Finally, raters independently scored 3 videos of subjects with varying degrees of movement compensations. When the rater scoring was deemed to be acceptably close to the trainer (± 10 points) they were allowed to score independently. If the score varied by more than ± 10 points (± 3% error on range of scale), the rater continued to review videos with the trainer. This process continued until the rater became independent. Once training was complete, each video was scored by 2 raters, if total scores differed by over ± 10 points, a third rater scored the video. Scores were averaged for use in the final analysis. Possible scores range from 0 to 261 points, with lower scores indicating fewer observed compensations, or better movement quality.

### Accelerometer variables

Data were extracted from bilateral, wrist worn accelerometers (wGT3X+, Actigraph, Pensacola, FL, USA) for 24 h at the selected time point matching the video that was scored. Accelerometers are a valid and reliable instrument to capture upper limb movement in daily life in individuals after stroke [16, 18, 27–30] and non-disabled adults [13, 14, 31].

For the selected time point, accelerometers were donned at the beginning of their session, prior to their in-clinic assessments and intensive upper limb therapy, then worn for an entire day afterward. Accelerometers were returned on the next treatment session and the data were downloaded using ActiLife 6 software (Actigraph Corp, Pensacola, FL, US). Raw data were sampled at 30 Hz. Data from the three 3 axes were filtered and converted to activity counts, where 1 count = 0.001664 g, using the proprietary algorithm. Data were then binned into 1-s epochs, and activity counts across each axis were combined creating a single vector magnitude value [17]. Using custom-written software in MATLAB (Mathworks Inc, Natick, MA, USA), ten variables were calculated for in-clinic and out-of-clinic time from the recorded data. Sleep was not excluded from the analysis, as persons with stroke have irregular sleep patterns which would prove challenging to extract definitive time for sleep from the data and inclusion of time sleeping does not change calculated variables [31, 32]. Recording time was separated into 1.5 h of in-clinic time which included upper limb assessments and intensive therapy (targeting repetitions of upper limb movement) and then 22.5 h of out-of-clinic wear. Note that movement compensation was not discouraged nor encouraged during the in-clinic or out-of-clinic time periods. Variables quantified different aspects of upper limb movement and can be conceptualized into variables measuring movement time, movement magnitude, and movement variability [13, 14, 18, 31, 33]. Table 1 provides a summary of variables. In addition, two newly proposed variables were calculated, the jerk asymmetry index [34] and the spectral arc length [35, 36]. These variables were calculated as they have been proposed to measure smoothness of movement, an aspect of quality of movement, by others in the field.

**Table 1:** Accelerometer variables

Variable name	Description
*Time*	
Isolated non-paretic limb activity [31]	Time, in hours, that the non-paretic limb is moving, while the paretic limb is still
Isolated paretic limb activity [31]	Time, in hours, that the paretic limb is moving, while the non-paretic limb is still
Bilateral activity [13, 31]	Time, in hours, that both upper limbs are moving together
Use ratio [16, 28, 47]	Ratio of hours of paretic limb movement, relative to hours of non-paretic limb movement
*Magnitude*	
Paretic limb magnitude [48, 49]	Magnitude of accelerations of the paretic limb, in activity counts*
Bilateral magnitude [13, 31]	Intensity, or magnitude of accelerations, of movement across both arms, in activity counts*
Magnitude ratio [13, 31, 49]	Ratio of the magnitude of paretic UL accelerations relative to the magnitude of the non-paretic UL accelerations. This ratio reflects the contribution of each limb to activity, expressed as a natural log
*Variability*	
Variability of paretic movement [48, 49]	Standard deviation of the magnitude of accelerations across the paretic limb, reflecting the variability of paretic limb movement, in activity counts*
Variability of bilateral movement [48, 49]	Standard deviation of the magnitude of accelerations across both limbs, reflecting the variability of bilateral upper limb movement, in activity counts*
Variation ratio [48, 49]	Ratio of the variability of paretic limb accelerations relative to the variability of the non-paretic limb accelerations, reflecting the relative variability in the paretic limb
*Smoothness*	
Unimanual Jerk Asymmetry Index [34]	Ratio of the average jerk magnitude between the paretic upper limb and the nonparetic upper limb. Higher jerk represents less smooth movement, and an index value of 0 represents similar smoothness of movement in the paretic and non-paretic limbs. Values are bounded between − 1 to + 1
Spectral arc length [35, 36]	A measure of movement smoothness that quantifies movement intermittencies independent of the movement’s amplitude and duration. Longer spectral arc lengths are reflective of less smooth or less coordinated movement

*Activity counts are computed by the Actilife proprietary software such that 1 activity count = 0.001664 g

### Analysis

All data were analyzed in R, an open source statistical computing program. The main analyses evaluated the relationships between the compensatory movement scores and each calculated accelerometer variable. Spearman rank correlations were chosen because relationships between compensatory movement scores and accelerometer variables were not assumed to be linear. Criteria for statistical significance was set at α < 0.05. The following criteria were used to interpret correlation coefficients: coefficients of rho ≥ 0.25 or below were considered low, coefficients ranging from 0.26 to 0.50 were considered moderate, coefficients from 0.51 to 0.75 were considered good, and those greater than 0.75 were considered excellent [37]. Beyond the individual relationship analysis, an exploratory, step-wise multiple regression evaluated how multiple accelerometer variables might collectively explain the variance in compensatory movement scores.

## Results

### Participants

Demographics of the participants are provided in Table 2 and have been reported elsewhere [20]. Overall, the sample had chronic upper limb paresis post stroke of mild to moderate severity. Compensatory movement scores were highly variable across the sample, with a mean of 73.7 ± 33.6, and a range from 11.5 to 188. This range indicates that none of the subjects were free from compensatory movements, and no subject used the maximum amount of compensations defined by the checklist. The majority of movement compensations were observed at the shoulder (28%). The second highest observed compensations were at the trunk (22%), followed by the fingers (21%), fluidity and movement precision (14%), elbow (5%), wrist (4%), head (3%), and finally forearm (2%).

**Table 2:** Characteristics of sample, values are means ± SD (range) or % of total sample unless otherwise specified

Descriptors (n = 78)	
Age (years)	61.9 ± 10.5 (32, 85)
Gender	35% Female 65% Male
Type of stroke	72% Ischemic 13% Hemorrhagic 15% Unknown
Ethnicity	99% Non-Hispanic/Latino 1% Hispanic/Latino
Months post stroke (median, min/max)	12, 5/221
Affected limb	46% Left 54% Right
% Concordance*	51%
% Independent with ADL	79%
Baseline ARAT Score	32.4 ± 11.2 (10–48)
Compensatory Movement Score	73.7 ± 33.6 (11.5–188)
Baseline use ratio	0.66 ± 0.23 (0.22–1.32)

*Concordance is the percent of individuals whose paretic UL was their dominant UL

### Relationships of variables to compensatory movement

**Fig. 1:**
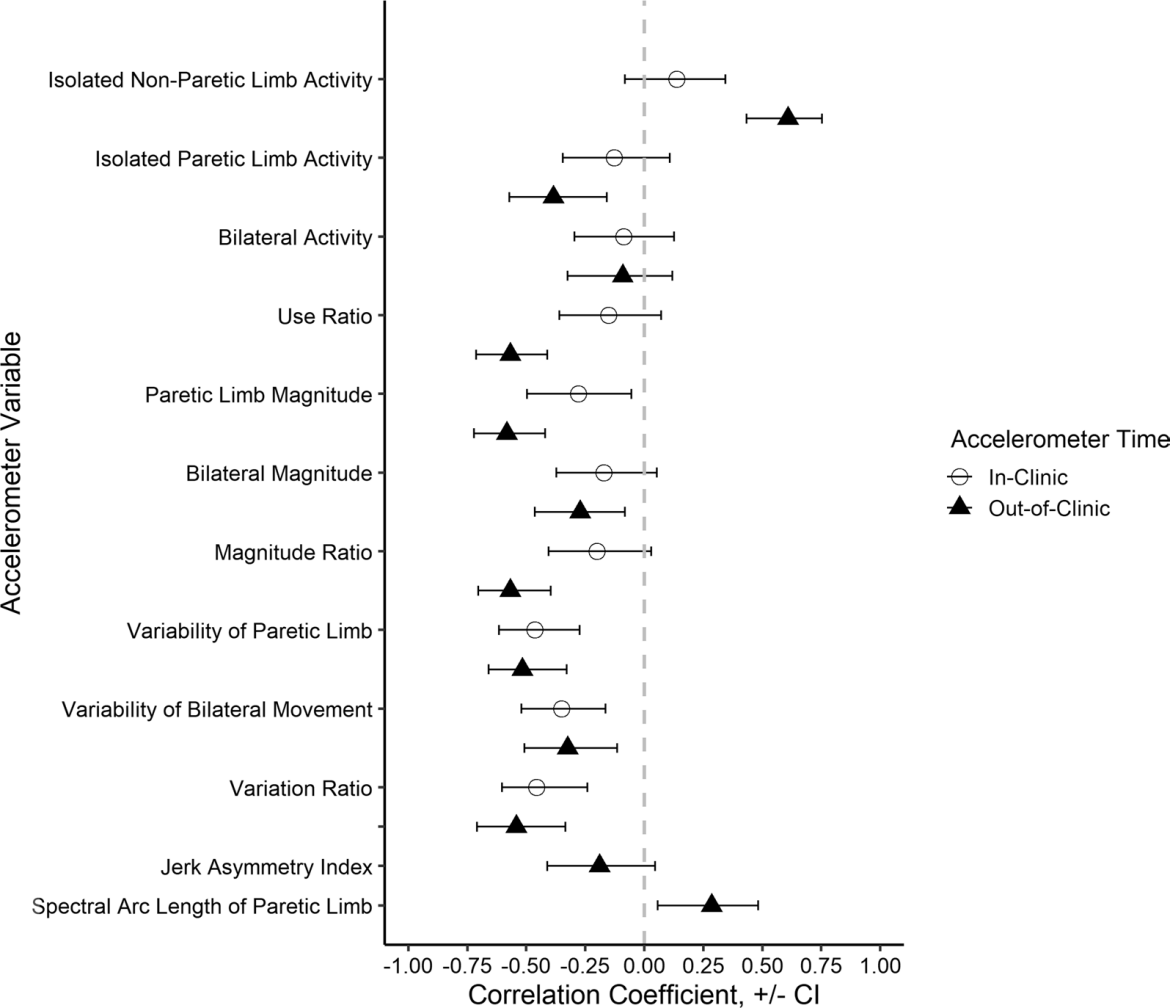
Relationships (x-axis) of compensatory movement scores to accelerometer variables (y-axis). Open symbols are in-clinic calculations, and closed symbols are out-of-clinic calculations. Error bars are 95% confidence intervals for each correlation coefficient. Lack of statistical significance occurs when error bars cross the vertical dashed line at 0

Overall, moderate correlations were observed between the compensatory movement scores and each accelerometer variable. Figure 1 shows the correlation coefficients and their 95% confidence intervals for each accelerometer variable, calculated from both in-clinic and out-of-clinic time. For most of the accelerometer variables, higher scores are better, making most of the correlation coefficients negative.

**Fig. 2:**
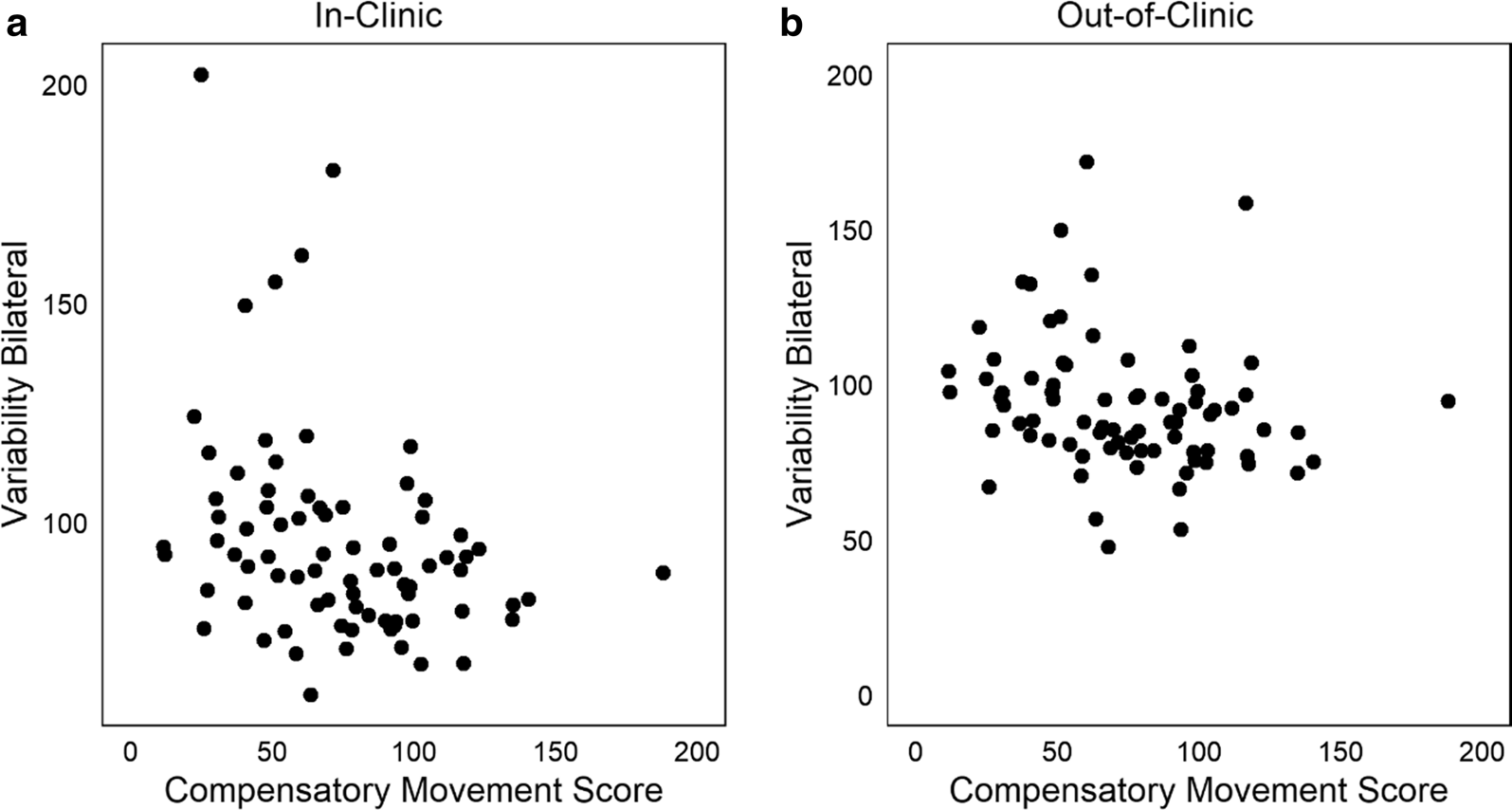
Relationship of variability of bilateral movement during in-clinic time (**a**, rho = − 0.32, p < 0.001) and out-of-clinic (**b**, rho = − 0.35, p < 0.01). This accelerometer variable had a similar moderate relationship both in and out-of-clinic

More than half of the accelerometer variables had similar relationships with compensatory movement scores when calculated from both in-clinic and out-of-clinic time. Figure 2 is a scatterplot of one such variable, variability of bilateral movement, where Fig. 2a illustrates its relationship to the compensatory movement score in-clinic (rho = − 0.35, p < 0.001), and Fig. 2b its relationship out-of-clinic (rho = − 0.32, p < 0.01). This moderate relationship indicates that individuals with more movement compensations tended to have less movement variability of the upper limbs, regardless of in which environment they were moving.

**Fig. 3:**
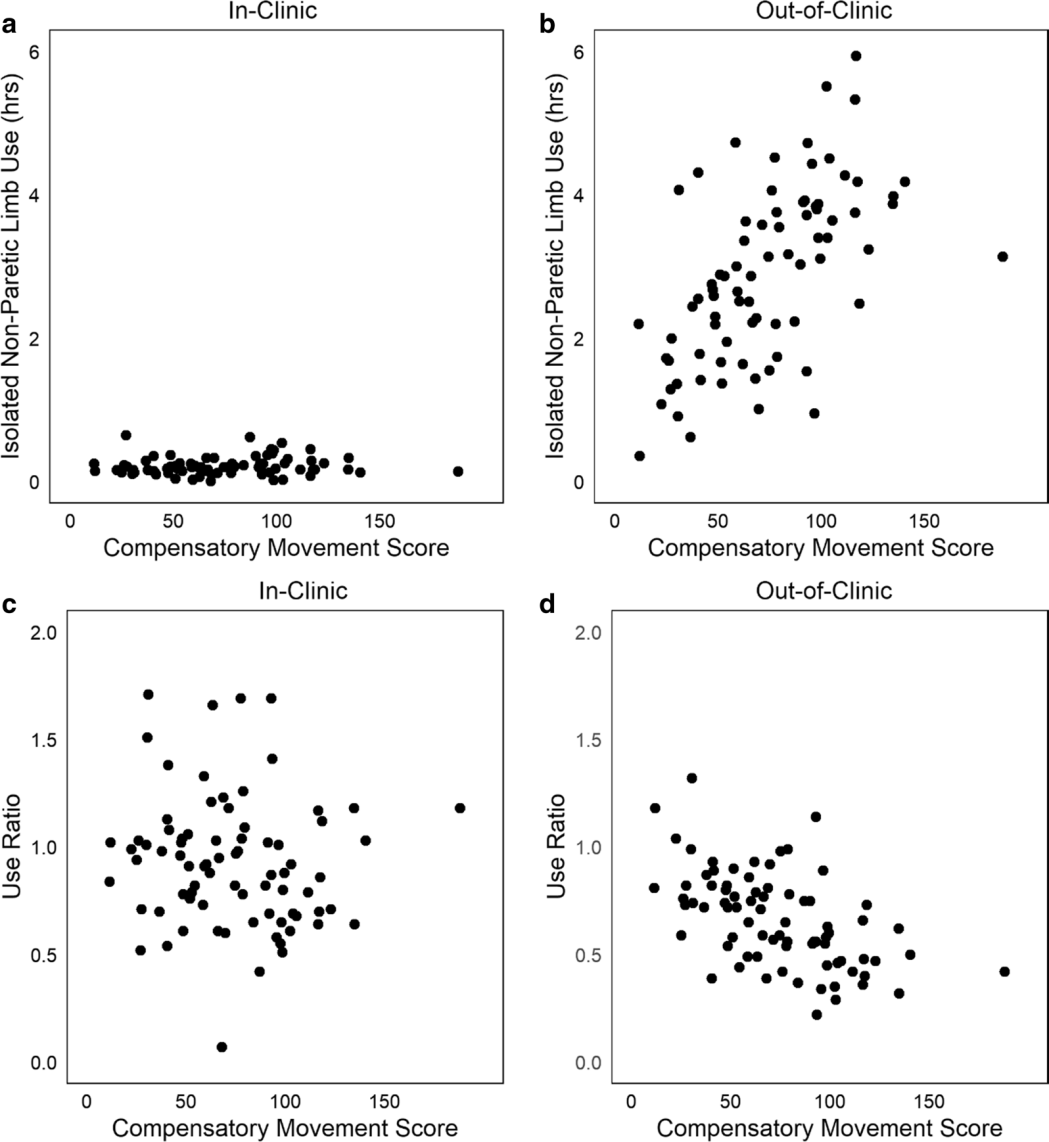
Relationship of isolated use of the nonpartetic limb to compensatory movement score, both in-clinic (**a**, rho = 0.14, p = 0.23) and out-of-clinic (**b**, rho = 0.61, p < 0.0001). Relationship of the use ratio to the compensatory movement score in-clinic (**c**, rho = − 0.15, p = 0.18) and out-of-clinic (**d**, rho = − 0.57, p = 0.18) These variables both had a little to no relationships in-clinic, yet good relationships out-of-clinic

Other accelerometer variables had a stronger relationship with compensatory movement scores, when calculated from time out-of-clinic versus in-clinic. Figure 3 shows scatterplots of two variables, isolated non-paretic limb activity and use ratio plotted relative to the compensatory movement score. Figure 3a illustrates the relationship of isolated non-paretic limb activity to compensatory movement score in-clinic (rho = 0.14, p = 0.23), and Fig. 3b its relationship out-of-clinic (rho = 0.61, p < 0.0001). The stronger positive relationship out-of-clinic indicates that individuals with more compensatory movements moved their non-paretic limb only more while out-of-clinic. The use ratio also had a stronger negative relationship with compensatory movement score out-of-clinic. Figure 3c illustrates the use ratio in-clinic to the compensatory movement score (rho = − 0.15 p = 0.18), and Fig. 3d its relationship out-of-clinic (rho = − 0.57, p < 0.0001). The strong relationship out-of-clinic indicates that, at home, individuals with more compensatory movements had a lower use ratio, indicating less relative paretic limb activity. None of the accelerometer variables had a stronger relationship during in-clinic time versus out-of-clinic time.

**Fig. 4:**
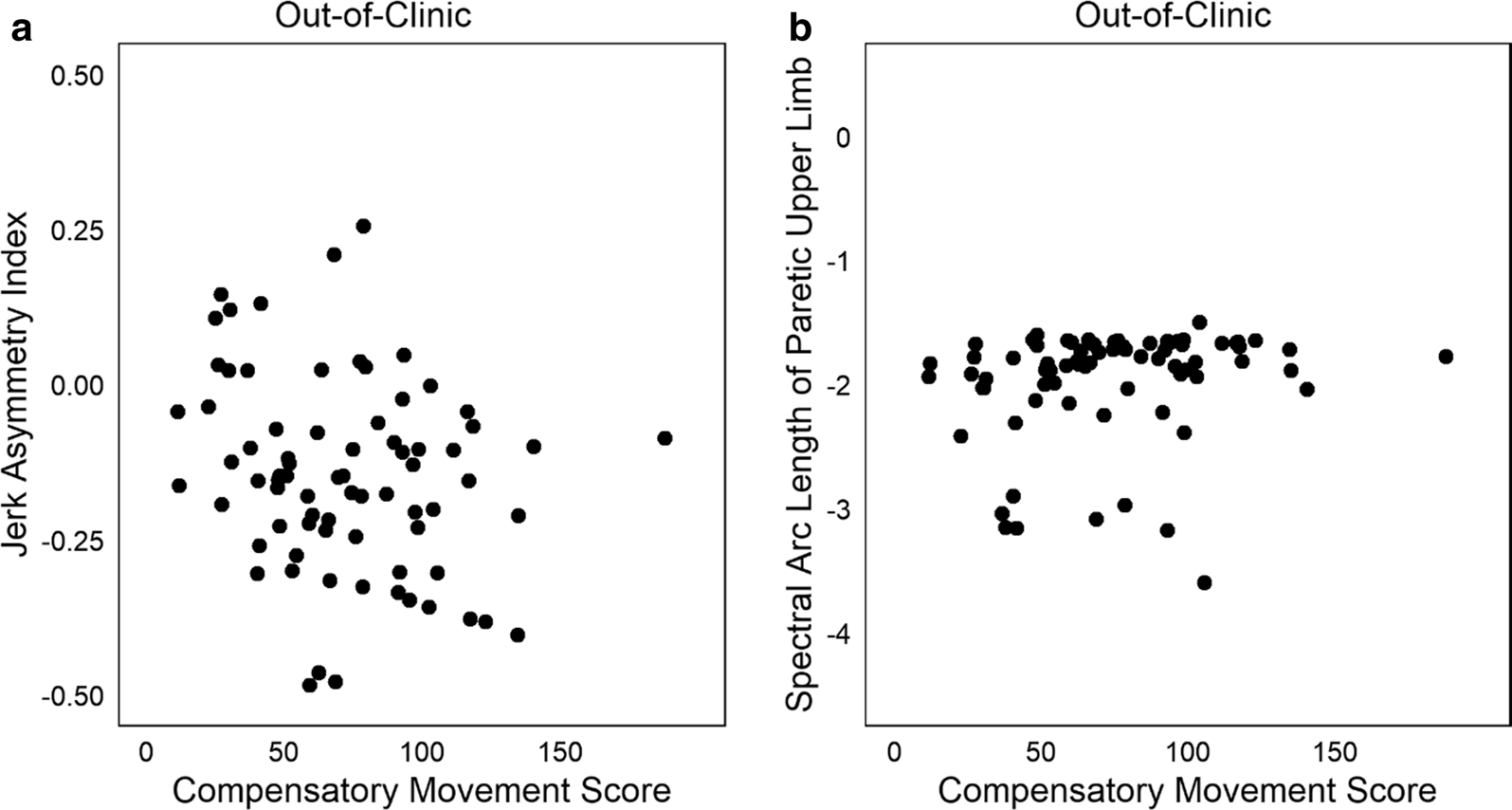
Relationship of two newly proposed metrics that quantify quality of upper limb movement. **a** Relationship of the Jerk Asymmetry Index to compensatory movement scores (rho = − 0.19, p = 0.09). **b** Relationship of the spectral arc length of the paretic limb to compensatory movement scores (rho = 0.29, p < 0.01). In **b**, one outlier with a spectral arc length of > − 6 has been omitted from the plot. Both variables are from out-of-clinic time and had a low relationship with the compensatory movement score

Two variables have been proposed to reflect movement smoothness as an aspect of quality of movement [34–36]. Figure 4 shows the relationship of the compensatory movement score to the jerk asymmetry index (Fig. 4a, rho = − 0.19, p = 0.09) and to the spectral arc length of the paretic limb (Fig. 4b, rho = 0.29, p < 0.01). Both variables had low relationships with the compensatory movement score.

Last, an exploratory multiple regression evaluated which combination of accelerometer variables explained the most variance in the compensatory movement score. Using a stepwise approach to select variables, two time-based variables explained the most variance. The use ratio out-of-clinic and the hours of isolated non-paretic limb use out-of-clinic together explained 37% of the variance in the compensatory movement score (R^2^ = 0.37, p ≤ 0.0001).

## Discussion

This study was a secondary analysis of an existing dataset that explored the relationships between accelerometer variables and compensatory upper limb movements in individuals with chronic hemiparesis. Individuals in the sample had a range of compensatory movements observed during the video scoring. Most accelerometer variables had a moderate relationship with the degree of compensatory movements of the upper limb for both in and out-of-clinic time points. This study used a novel approach to quantify compensatory movement patterns of the limb at a single time point, during completion of a standardized assessment. The scoring employed here could be a useful tool for other research studies, but will need substantial validation before it could be deployed clinically. Overall, these results indicate that accelerometry variables, while measuring movement quantity, can also reflect the use of general compensatory movement patterns of the upper limb.

Most accelerometer variables had moderate relationships with compensatory movement scores. The variables more strongly associated with compensatory upper limb movements quantified time, magnitude, and variability while participants engaged in activity out-of-clinic. For example, the strong relationship of movement compensations to isolated use of the non-paretic limb aligns with clinical expectations [3, 13, 38] that individuals who have more movement compensations of the paretic upper limb, frequently use their non-paretic limb to complete daily tasks at home. Since movement compensations typically add in movements at alternative joints (e.g. extra trunk flexion), it may be that the inefficiency of the compensatory movements leads the individual to instead use the non-paretic limb. Likewise, individuals who use more movement compensations have less variability in both paretic and bilateral limb movements. In general, reduced movement variability is considered to align with “an unhealthy pathological state or an absence of skill.” [39] Individuals who use more compensatory movements have fewer options for movement available [40, 41].

Some accelerometer variables tended to be have stronger relationships with compensatory movement scores when quantified from out-of-clinic recordings vs. in-clinic recordings. This is illustrated visually in Fig. 1, where more closed triangles are further from the zero line than open circles are. The in-clinic recordings here are from participation in an intensive, progressive, upper limb trial, where individuals are trained to use their affected paretic limb for functional activities [20]. Weaker relationships of some variables in-clinic confirms that therapy sessions were promoting activity of the affected upper limb. We note that the intent of the training protocol was to improve upper limb functional capacity, not to reduce movement compensations [20]. During in-clinic recordings, the accelerometer variables measure what an individual does during the training protocol. The out-of-clinic time measures how an individual moves their upper limbs during daily life [29, 42]. Based on the moderate or strong relationships, out-of-clinic, accelerometer variables reflect not just quantity of upper limb movement, but also collective use (more vs. less) of compensatory movements of the upper limb.

## Limitations

Several limitations should be considered when interpreting these data. First, video recordings of a standardized assessment were used to quantify movement compensations as a proxy for compensatory movements that would occur throughout the recording period. Given that research and therapy participants often try to do their best on tests in front of an assessor [31, 43–45], using these videos to quantify compensatory movements may be an under-estimate of the compensatory movements participants engage in throughout the day. Second, video-recording of the assessment was chosen to quantify compensatory movements over the video-recording of the therapy session. This decision was made because the assessment was the same for all, while the therapy sessions involved individualized therapeutic activities of different amounts, i.e. making it hard to compare across subjects. While the ARAT standardized assessment captures most upper limb movement components [46], one cannot rule out the possibility that alternative compensations might be observed within the therapy session or at home. Collectively these two limitations mean that we may have underestimated upper limb compensatory movements, and perhaps also underestimated the strength of the relationships of the accelerometer variables to the compensatory movement score. A third limitation is the use of coding from videos instead of using kinematic analysis of movement compensations [12]. Kinematic data from this sample does not exist. It is anticipated that using a kinematic analysis would not diminish the relationships of accelerometer variables to movement compensations of the upper limb, rather future studies using kinematics could be used to validate the relationships found here. Additionally, kinematic analysis could expand upon those relationships by indicating the specific movement compensations an individual is using with their upper limb, not just the general quantification used here. In the future kinematics might be captured with accelerometry, if sensors were cheaper and, smaller, and wearing multiple sensors over hours was not too burdensome.

## Conclusions

This study quantified movement compensations of the upper limb and determined their relationship to accelerometer variables. Individuals who move their limbs more in daily life with respect to time and variability tend to move with less movement compensations and more normal movement patterns. Likewise, individuals who move their paretic limbs less and their non-paretic limb more in daily life tend to move with more movement compensations at all joints in the paretic limb and less normal movement patterns. These results suggest that, for people with upper limb paresis due to chronic stroke (> 6 months), movement quality is not an independent construct from movement quantity. While accelerometers as a tool can reflect some information on movement quality, likely due to the association of movement quality with quantity, more work is needed to improve the methodology.

## Supplementary information

**Additional file 1: Table.**The suppplemental table is the compensatory movement scoring checklist. Items to create this checklist were synthesized from the following assessments, Reaching Performacne Scale (1), Upper Extremity Fugl- Meyer (2), Wolf Motor Function Test, (3) Action Research Arm Test, (4) Chedoke McMasters, (5) Stroke Rehabiliation Assessment of Movement (STREAM), (6) Motor Evaluation Scale for Upper Extremity in Stroke Patients (MESUPES), (7) Motor Assessment Scale (MAS), (8) and Quantative assessment of upper extremity function. (9) Compensatory behaviors on the checklist included movements at the head, trunk, shoulder,elbow, forearm, wrist, fingers and fluidity/movement. Compensations were scored as present or absent from the videotaped completion of the ARAT.

## Data Availability

Upon acceptance to the journal the data will be uploaded to NIH.Figshare.com. Data included will be individual compensatory movement score and extracted calculated accelerometer variables for both in-clinic and out-of-clinic time.

## Abbreviations

ADL: Activities of daily living
ARAT: Action Research Arm Test
NIHSS: National Institutes of Health Stroke Scale
