# Supplementary material for: Relationships between accelerometry and general compensatory movements of the upper limb after stroke
Source: J Neuroeng Rehabil. 2020 Oct 20;17:138. doi: 10.1186/s12984-020-00773-4 (PMC7576735; doi:10.1186/s12984-020-00773-4)
Supplement: Supplementary file 1 — Additional file 1: Table.The suppplemental table is the compensatory movement scoring checklist. Items to create this checklist were synthesized from the following assessments, Reaching Performacne Scale (1), Upper Extremity Fugl- Meyer (2), Wolf Motor Function Test, (3) Action Research Arm Test, (4) Chedoke McMasters, (5) Stroke Rehabiliation Assessment of Movement (STREAM), (6) Motor Evaluation Scale for Upper Extremity in Stroke Patients (MESUPES), (7) Motor Assessment Scale (MAS), (8) and Quantative assessment of upper extremity function. (9) Compensatory behaviors on the checklist included movements at the head, trunk, shoulder,elbow, forearm, wrist, fingers and fluidity/movement. Compensations were scored as present or absent from the videotaped completion of the ARAT. [file 12984_2020_773_MOESM1_ESM.docx]

Relationships between accelerometry and general compensatory movements of the upper limb after stroke.

Barth J, Klaesner JW, and Lang CE

The additional table is the compensatory movement scoring checklist. Items to create this checklist were synthesized from the following assessments, Reaching Performacne Scale (1), Upper Extremity Fugl- Meyer (2), Wolf Motor Function Test, (3) Action Research Arm Test, (4) Chedoke McMasters, (5) Stroke Rehabiliation Assessment of Movement (STREAM), (6) Motor Evaluation Scale for Upper Extremity in Stroke Patients (MESUPES), (7) Motor Assessment Scale (MAS), (8)and Quantative assessment of upper extremity function.(9) Compensatory behaviors on the checklist included movements at the head, trunk, shoulder,elbow, forearm, wrist, fingers and fluidity/movement. Compensations were scored as present or absent from the videotaped completion of the ARAT.

1. Levin MF, Desrosiers J, Beauchemin D, Bergeron N, Rochette A. Development and validation of a scale for rating motor compensations used for reaching in patients with hemiparesis: the reaching performance scale. Physical therapy. 2004;84(1):8-22.

2. Fugl-Meyer AR, Jääskö L, Leyman I, Olsson S, Steglind S. The post-stroke hemiplegic patient. 1. a method for evaluation of physical performance. Scandinavian Journal of Rehabilitation Medicine. 1975;7(1):13-31.

3. Wolf SL, Catlin PA, Ellis M, Archer AL, Morgan B, Piacentino A. Assessing Wolf motor function test as outcome measure for research in patients after stroke. Stroke. 2001;32(7):1635-9.

4. Lyle. A performance test for assessment of upper limb function in physical rehabilitation treatment and research. International journal of rehabilitation research. 1981;4(4).

5. Gowland C, Stratford P, Ward M, Moreland J, Torresin W, Van Hullenaar S, et al. Measuring physical impairment and disability with the Chedoke-McMaster Stroke Assessment. Stroke. 1993;24(1):58-63.

6. Daley K, Mayo N, Wood-Dauphinée S. Reliability of scores on the Stroke Rehabilitation Assessment of Movement (STREAM) measure. Physical therapy. 1999;79(1):8-23.

7. Van de Winckel A, Feys H, van der Knaap S, Messerli R, Baronti F, Lehmann R, et al. Can quality of movement be measured? Rasch analysis and inter-rater reliability of the Motor Evaluation Scale for Upper Extremity in Stroke Patients (MESUPES). Clinical rehabilitation. 2006;20(10):871-84.

8. Carr JH, Shepherd RB, Nordholm L, Lynne D. Investigation of a new motor assessment scale for stroke patients. Physical therapy. 1985;65(2):175-80.

9. Carroll D. A quantitative test of upper extremity function. Journal of Chronic Diseases. 1965;18(5):479-91.
